# Supplementary material for: Towards a new approach to reveal dynamical organization of the brain using topological data analysis
Source: Nat Commun. 2018 Apr 11;9:1399. doi: 10.1038/s41467-018-03664-4 (PMC5895632; doi:10.1038/s41467-018-03664-4)
Supplement: Supplementary file 1 — Supplementary Information(PDF 9415 kb) [file 41467_2018_3664_MOESM1_ESM.pdf]

Supplement to: Towards a new approach to visualize and quantify brain's dynamical organization using topological data analysis

Manish Saggar[1*], Olaf Sporns[2], Javier Gonzalez-Castillo[3], Peter A. Bandettini[3,4], Gunnar Carlsson[5,6], Gary Glover[7], Allan L. Reiss[1,7]

[1]Department of Psychiatry & Behavioral Sciences, Stanford University
[2]Department of Psychological & Brain Sciences & Network Science Institute, Indiana University
[3]Section on Functional Imaging Methods, National Institute of Mental Health, NIH
[4]Functional MRI Core, National Institutes of Mental Health
[5]Department of Mathematics, Stanford University
[6]Ayasdi, Inc.
[7]Department of Radiology, Stanford University

*Corresponding Author:
Manish Saggar, Ph.D. (email: saggar@stanford.edu)

Supplementary Figures and Tables

In this supplement we provide figures and tables as supplementary information for the main text. Specifically, we have added figures to provide (1) better intuition behind Topological Data Analysis (Supplementary Figure 1); (2) more information about the web-based interface to interact with the TDA-generated shape graphs (Supplementary Figures 2-4); (3) validation and reliability analyses (Supplementary Figures 5, 6, 7, 9, 11, and 12); and (4) parameter perturbation analysis (Supplementary Figure 8 and 10). We also include a supplementary table to provide GLM results from the analysis run to examine the neurophysiological basis of coreness in the shape graphs (Supplementary Table 1).

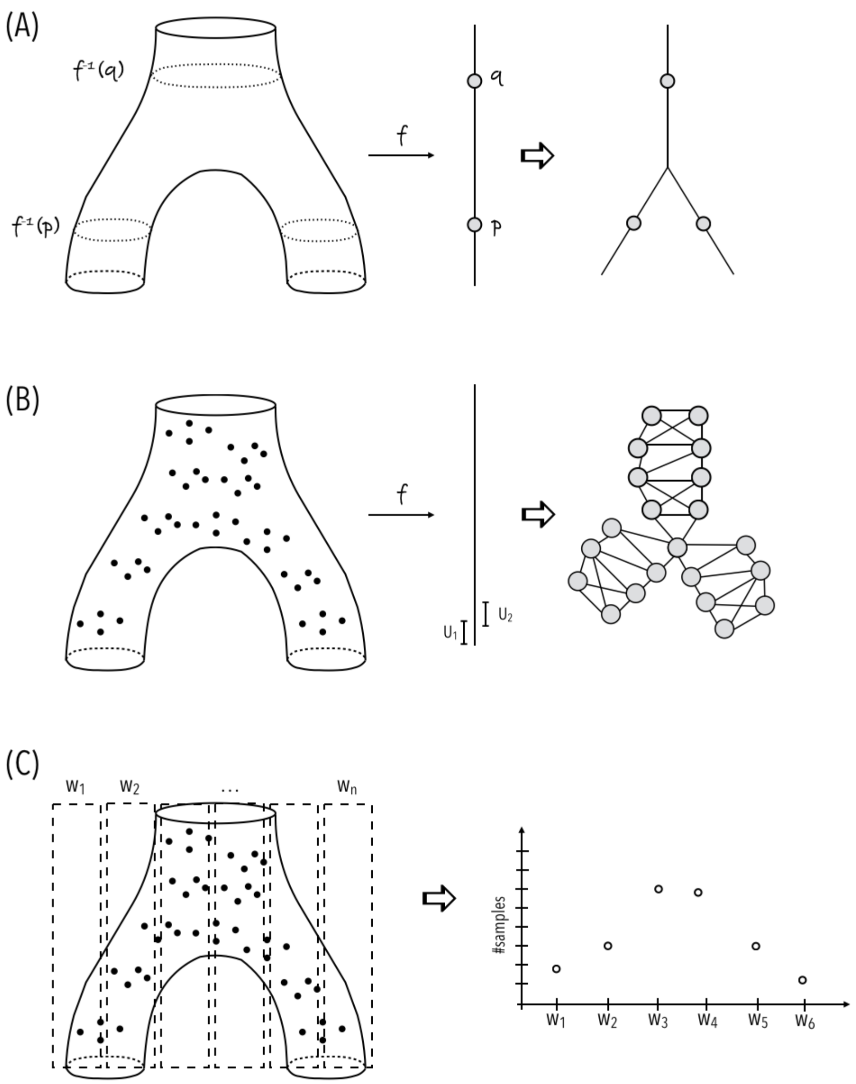

Supplementary Figure 1: Application of TDA-based Mapper to examine the shape of a toy 3D dataset (a pair of pants). (A) Shows an ideal situation, where we have a fully sampled dataset. Hence, if we simply take a geometric filter (f) that counts the number of connected components (e.g., for a point p in the domain of f, $f^{-1}(p) = 2$; and for another point q, $f^{-1}(q) = 1$), we can create a representation of the original dataset that looks like an inverted 'Y'. This representation provides a unique insight that our data has a two-legged structure. (B) Shows a more realistic situation, where we could only sample subparts of the dataset (shown as black dots inside the pair of pants). Here, Mapper uses three additional steps after filtering, namely, binning, partial clustering, and creating a combinatorial representation (a.k.a. shape graph). It is important to note that the resulting graph, although a simplicial complex, can still reveal the insight that the data has a two-legged structure. Further, the resulting graph is invariant to coordinate deformations (e.g., rotation) or noise deformations (e.g., missing parts of data). Lastly, the resulting graph can be examined to better understand the shape of the original data (e.g., detect cores or communities in the shape graph). (C) Shows a traditional window-based analysis over time. The $W_i s$ represent consecutive temporal windows selected for dynamical analysis. For simplicity, we count the number of samples in each window. This results in a plot of changing #samples over windowed periods of time. While this temporal representation simplifies the data, it does not reveal the true intrinsic structure or shape of the data.

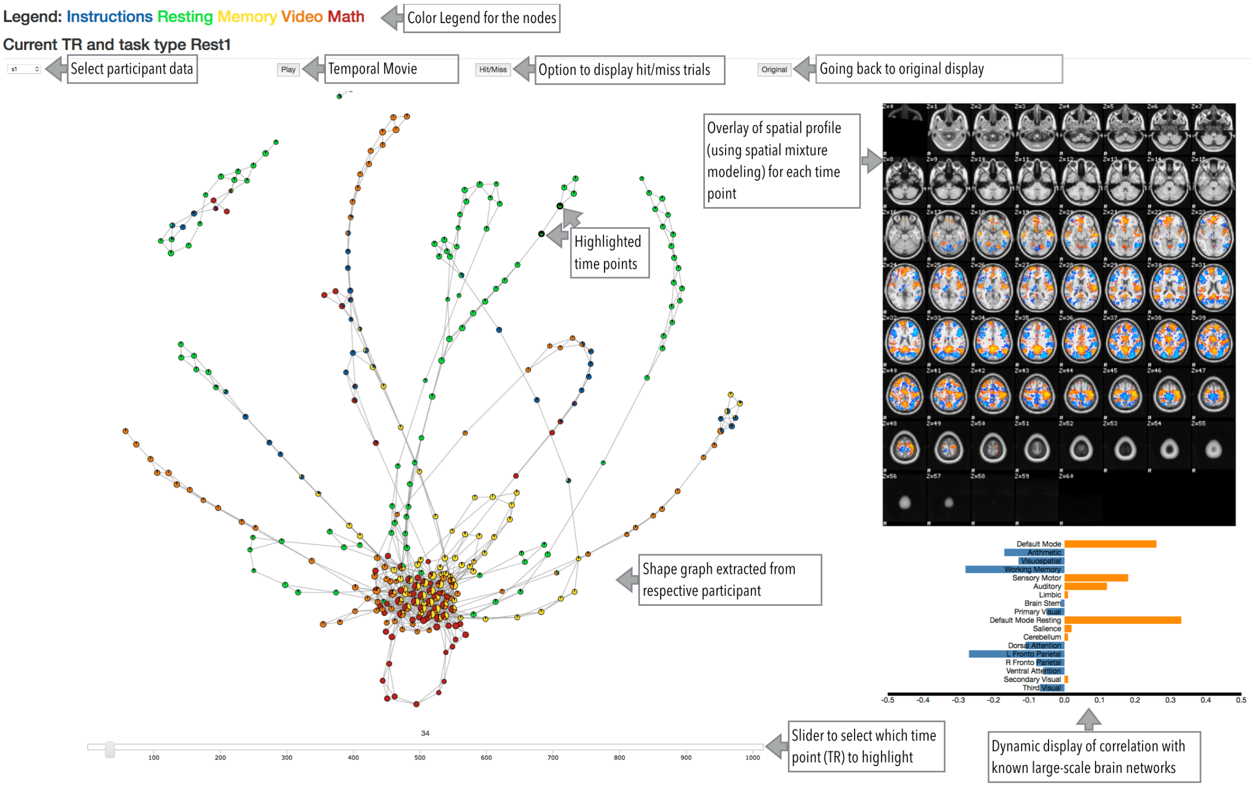

Supplementary Figure 2: Web-based interface to interact with the shape graph and display its properties interactively. This figure is a screenshot of the interface, with labeled boxes detailing each aspect of the interface. In this view, the nodes in the shape graph are annotated using task type of the encompassing time points.

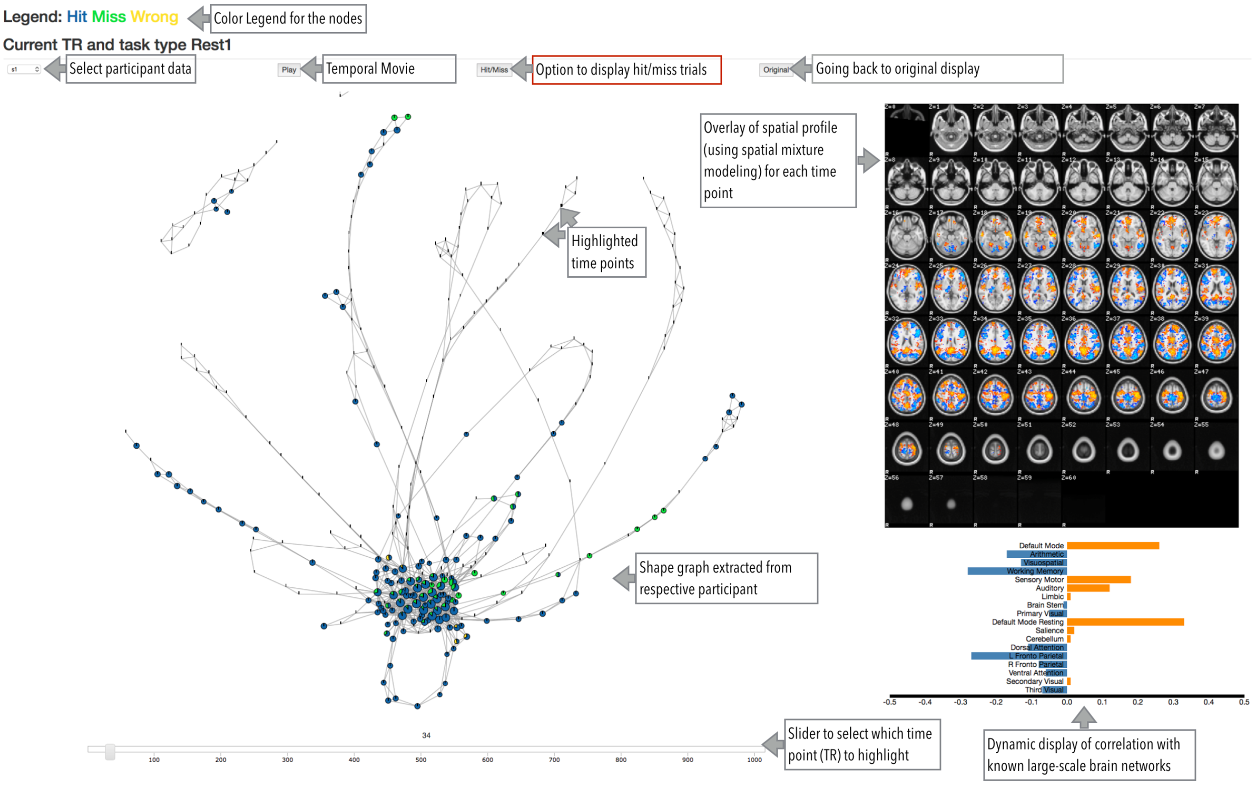

Supplementary Figure 3: Web-based interface to interact with the shape graph and display its properties interactively. Screenshot of the interface with labeled boxes shown here. In this view, the shape graph is annotated based on whether the encompassing time-points in each node represent a hit or miss trial.

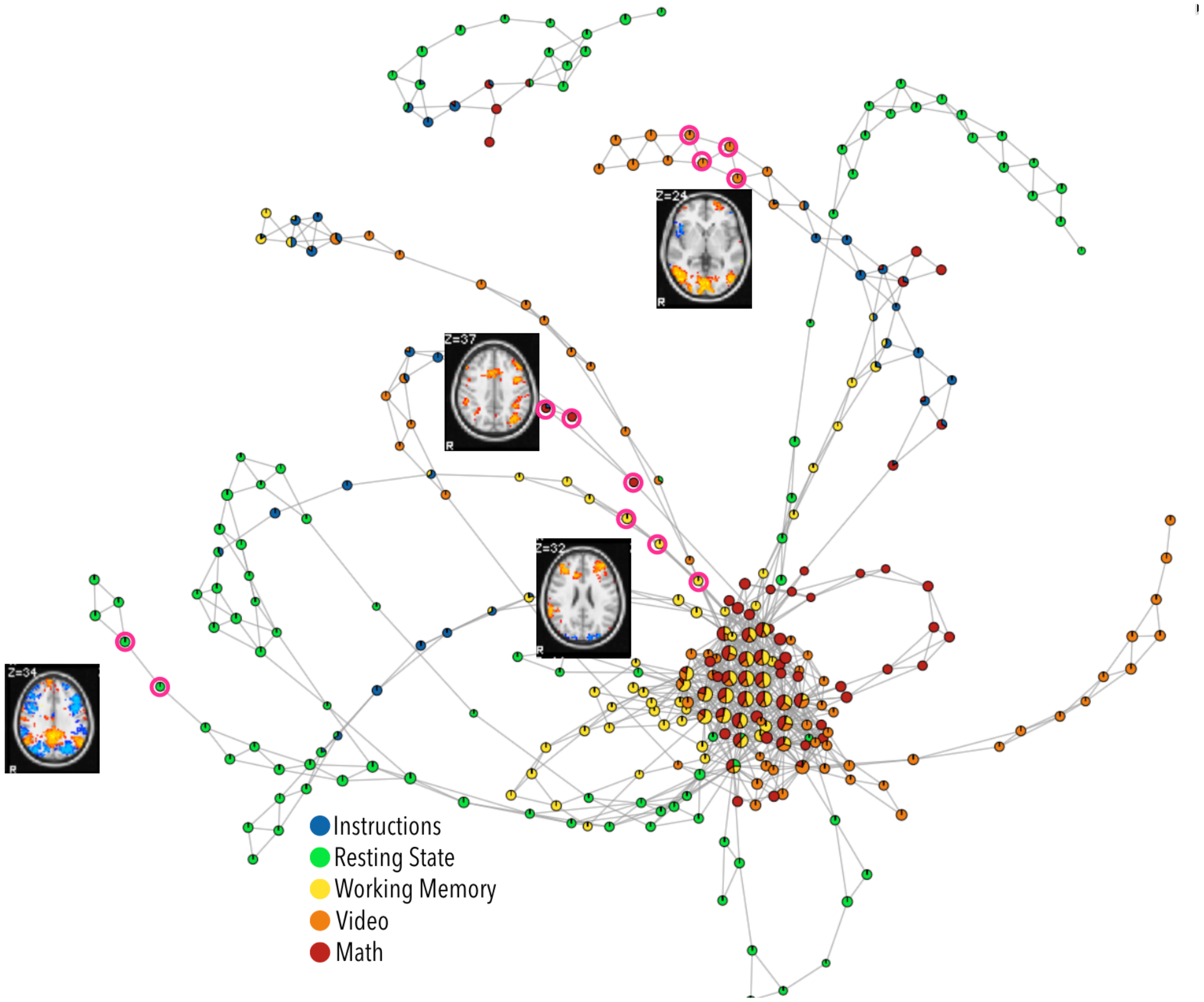

Supplementary Figure 4: To reveal the spatial profile of different nodes in the shape graph, we used Spatial Mixture Modeling (SMM[1]). Thus, for each node and the included time frames, a normalized statistical map was generated and fed to the SMM algorithm to generate activation (shown in red-yellow color scale) and deactivation (blue-white color scale) maps. Here we show spatial profiles for a small sample of nodes (highlighted in pink colored circle) from the shape graph of one of the representative participants (see Supplementary Movie 1 for detailed transitions and associated spatial profiles over time). As evident, although with low SNR, the associated spatial profile for different task nodes resembles the regions typically activated (on average across the scan/block) by the respective tasks.

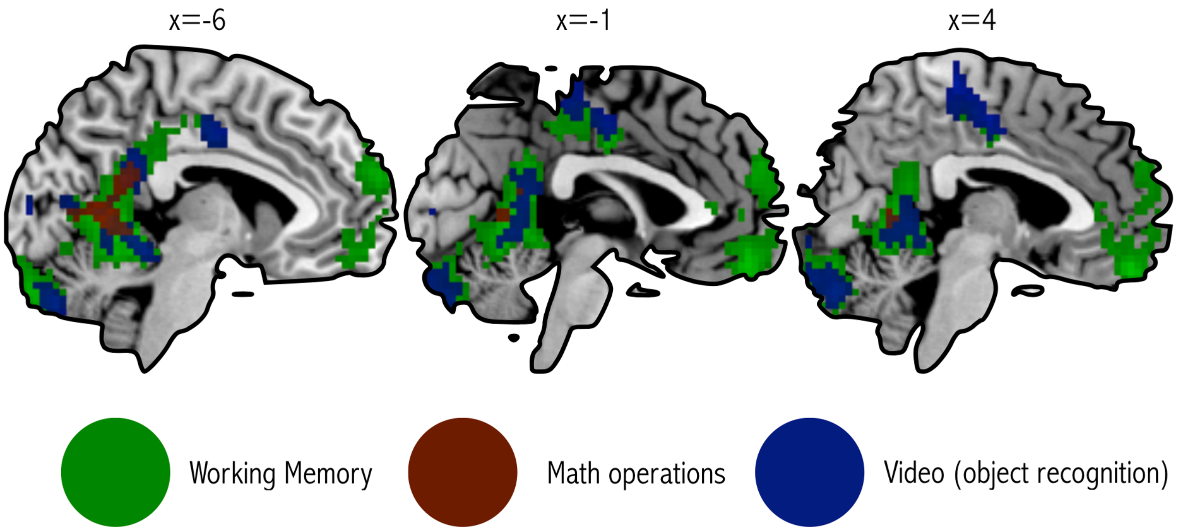

Supplementary Figure 5: Shows the significant brain activation maps for the brain regions that were negatively associated with the coreness scores (CS), within each cognitive task. To better depict the overlap across the three tasks, the maps are thresholded and binarized at cluster correction FWER $p<0.05$ using $Z>2.3$. Across the three cognitive tasks we found a negative association with CS in the posterior cingulate cortex region (PCC). This overlap suggests that lower CS (representing peripheral nodes) were associated with task-nonspecific brain activation in the regions usually linked with intrinsic processing at rest (or the default mode network).

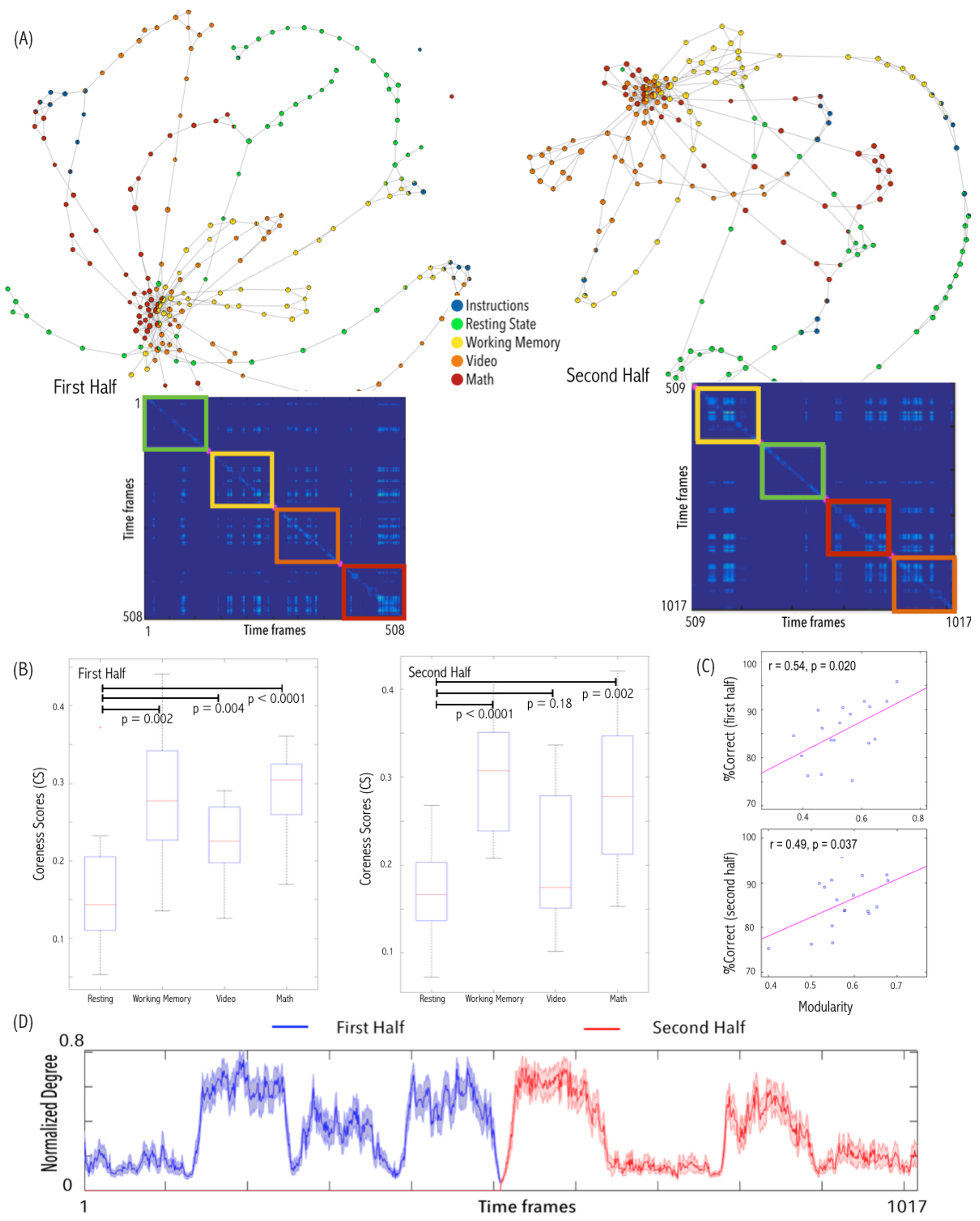

Supplementary Figure 6: Reliability analysis by splitting the data into two halves. (A) Depicts the shape and its Temporal Connectivity Matrix (TCM) extracted using first and second half of the data independently, for a representative participant (S01). (B) Shows the boxplot of coreness score for both halves. The resting state is still in the periphery (i.e., low coreness score), while other cognitively demanding states are part of the core (i.e., high coreness score). Box-plot depicting coreness across all tasks (for CMP participants (n=18)). One-way ANOVA was run to determine the effect of task on coreness scores (First Half: $F(3,51)=12.21$, $p<0.0001$ and Second Half: $F(3,51)=10.25$, $p<0.0001$), followed by post hoc two-sided t-tests comparing rest to other tasks (*First Half:* Rest vs. Memory [$t(17)=-3.691$, $p=0.002$], Rest vs. Video [$t(17)=-3.347$, $p=0.004$] and Rest vs. Math [$t(17)=-4.814$, $p<0.0001$]; *Second Half:* Rest vs. Memory [$t(17)=-5.184$, $p<0.0001$], Rest vs. Video [$t(17)=-1.4$, $p=0.18$], and Rest vs. Math [$t(17)=-3.71$, $p=0.002$]). (C) Significant correlations were observed between task performance (%correct) averaged over the three cognitive tasks and modularity scores (Q_{mod}), separately for each half. (D) Presents average temporal transitions over all participants for the first (blue) and second (red) halves.

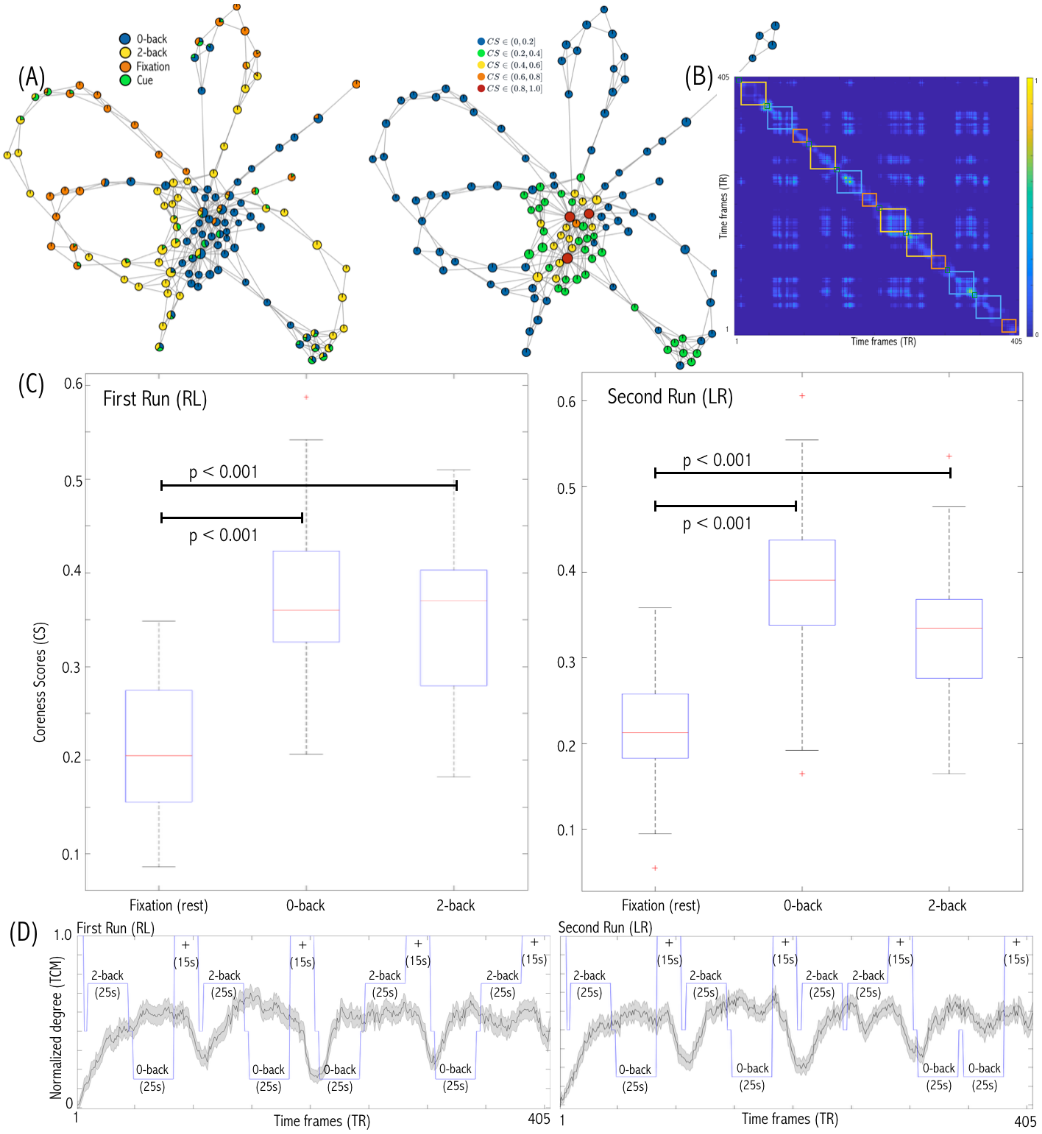

Supplementary Figure 7: Results from the HCP data. The shape graphs were generated from a working memory task. (A) Shows one shape graph annotated in two different ways from a representative participant. The first shape graph is annotated using condition-type as colors of the node, while the second shape graph is annotated using coreness scores. (B) Shows the Temporal Connectivity Matrix (TCM) extracted from one of the representative participants. (C) Shows the coreness score (boxplot over all participants; n=38). The resting state (or between-task fixation) was observed to have low coreness score, while other cognitively demanding tasks are part (or closer) to the core (denoted by high coreness scores). One-way ANOVA revealed the effect of task on coreness scores for both runs (First Run: $F(2,74)=28.89$, $p<0.0001$; and Second Run: $F(2,74)=31.03$, $p<0.0001$), followed by post hoc two-sided t-tests comparing rest to other tasks (First Run: Rest vs. Zero-back [$t(17)=-7.76$, $p<0.0001$] and Rest vs. 2-back [$t(17)=-6.01$, $p<0.0001$]; and Second Run: Rest vs. Zero-back [$t(17)=-7.58$, $p<0.0001$] and Rest vs. 2-back [$t(17)=-6.08$, $p<0.0001$]). (D) Presents average temporal transitions across all participants, separately for each run. It is important to note that in contrast to the CMP data, here, we show transitions within a task. The experimental paradigm is overlaid in blue colored line and we have annotated different conditions (and duration in s) as legends. Please also note that the TR for HCP data is much faster than the CMP data $TR_{HCP} = 0.72$ s and $TR_{CMP} = 1.5$ s).

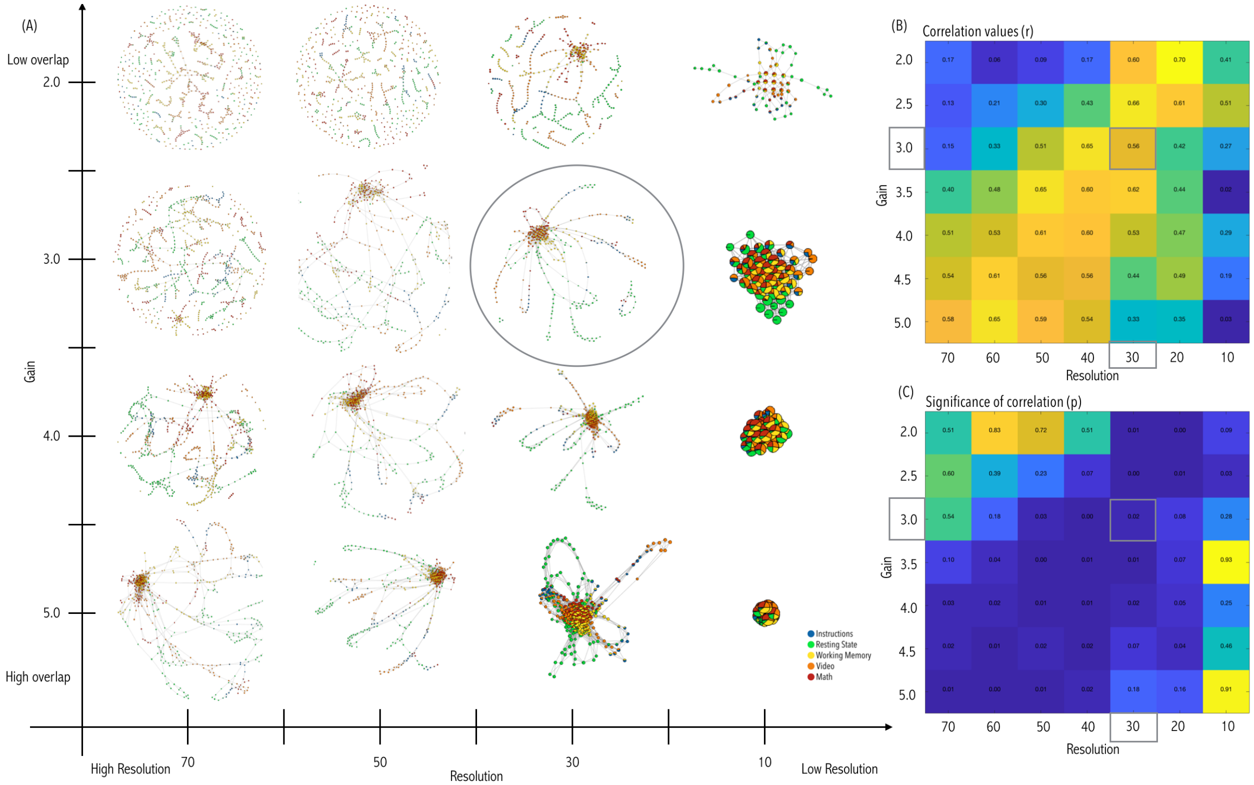

Supplementary Figure 8: Perturbation of TDA parameters and its effect on shape graphs and association with behavioral performance. (A) Depicts the TDA-derived shape graphs for 16 (out of 49) different combinations of the two parameters (Resolution and Gain) for one representative participant (S01). The core-periphery structure was observed in a majority of the parameter combinations. We have highlighted (circled) the shape graph derived using the data-driven approach of finding TDA parameters (Resolution = 30, Gain = 3). (B) Heat map (with labels denoting the values) of correlation values between modularity of shape graphs and behavioral task performance (%correct) for all 49 combinations of TDA parameters. As evident, the correlation is robust in the face of parameter perturbation. (C) Heat map of p-values of correlation between modularity of shape graphs and behavioral task performance (%correct), for all 49 combinations of TDA parameters.

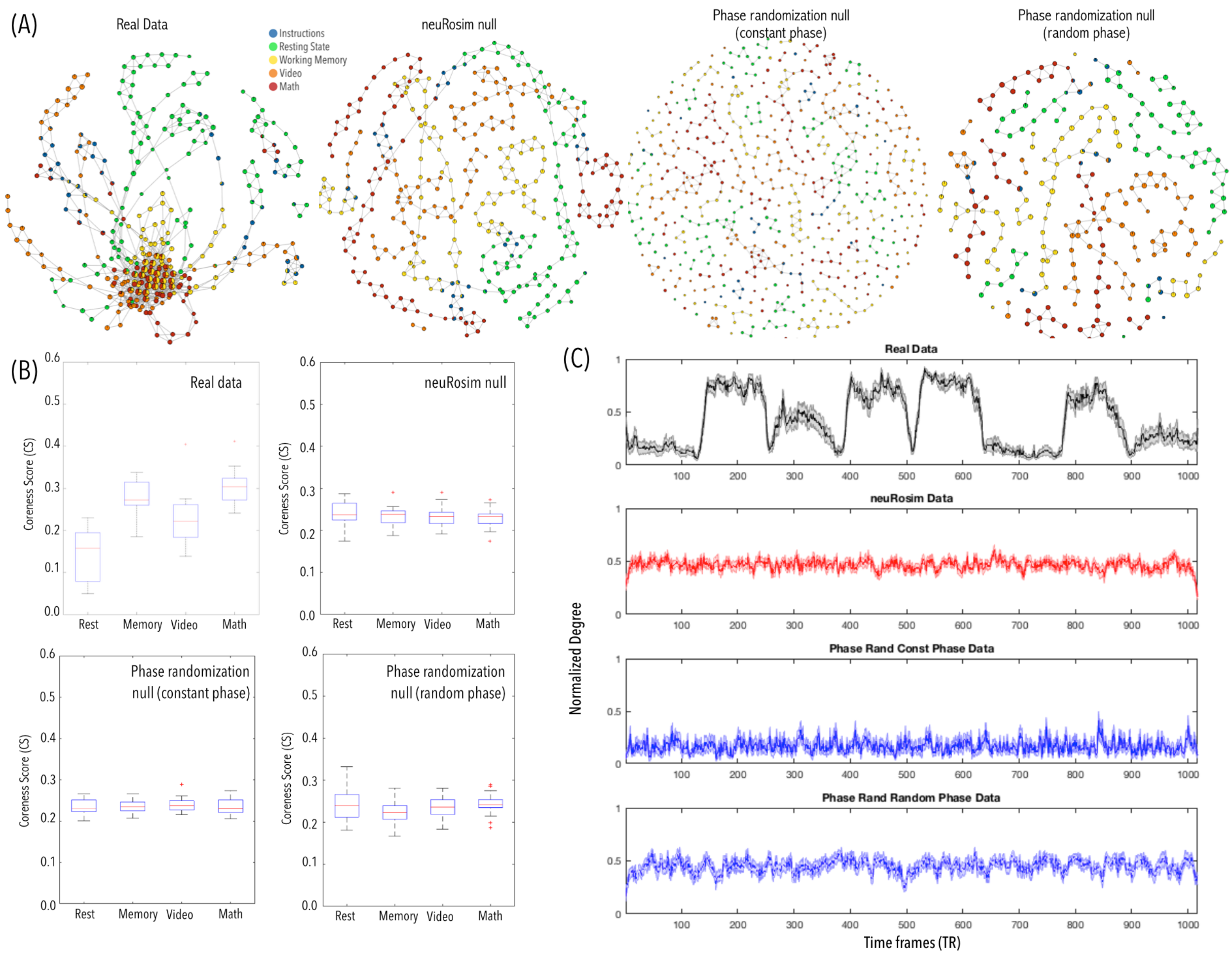

Supplementary Figure 9: Validation analysis using three different null models of the CMP dataset. (A) Shows a representative shape graph generated from real data versus three different shape graphs generated using the null models for the same representative participant. As evident, the shapes derived from null models have no core-periphery structure. The nodes are mostly linearly linked, likely due to the modeled spatiotemporal autocorrelation in the null data. (B) The coreness score for each task type for the three null models, as compared to the real data set. As evident, the coreness scores derived from null models have no variation of coreness scores across tasks. Box-plots depicting "coreness" across all tasks (null models, n=18)). One-way ANOVA was run to determine the effect of task on coreness scores (neuRosim null: $F(3,51)=0.398$, $p=0.76$; phase randomization (constant phase): $F(3,51)=0.363$, $p=0.78$; and phase randomization (random phase): $F(3,51)=1.01$, $p=0.394$) (C) Shows the normalized degree of TCM for all three null models. Averaged normalized degree of TCM nodes from the real data is also shown (in black) for comparison. As clearly shown, the transitions between different tasks blocks, which were evident from the real data, are not present in any of the null models.

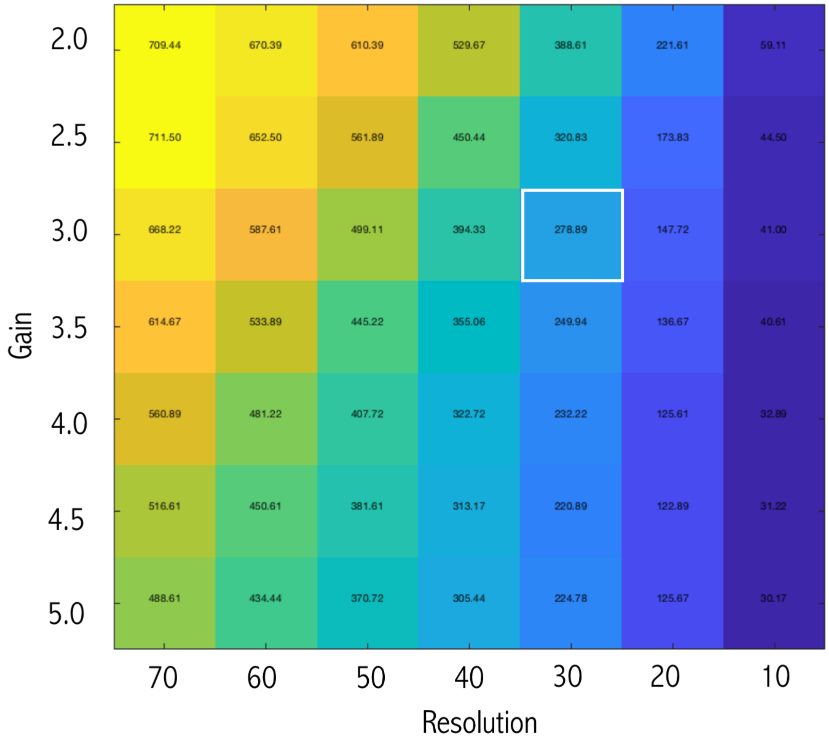

Supplementary Figure 10: Effect of parameter perturbation on the size of shape graph in CMP participants. The heat map shows the effect of varying parameter values (Gain and Resolution) on the average size of shape graph (i.e., number of nodes). The data originally had 1,017 time frames and the TDA-generated shape graphs can compress the representation by almost a factor of 4 (as shown in the highlighted square for parameters chosen by the data-driven approach G=3.0 and R=30).

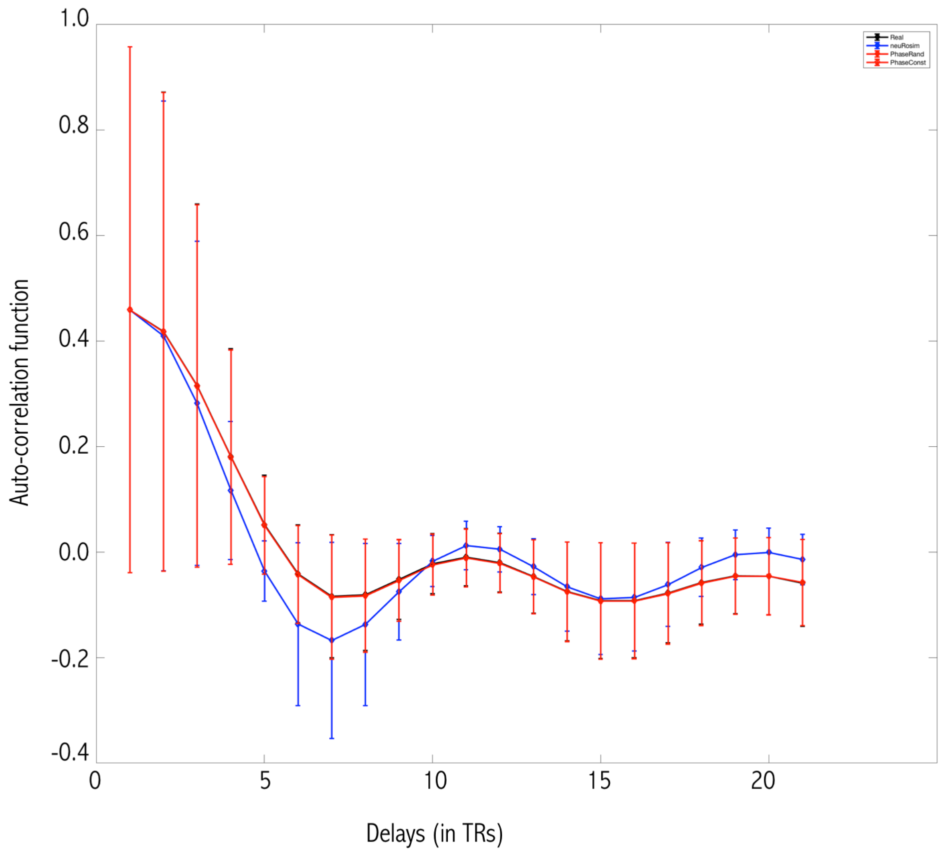

Supplementary Figure 11: The averaged autocorrelation function derived from one representative participant (S01) for the real data (in black) and three null models (neuRosim in blue color, Phase randomization with constant phase in red diamonds and with random phase in red circles). The error bar represents standard deviation of the mean (over voxels from the gray matter). As evident, the autocorrelation function of the null models closely follows that of the real data.

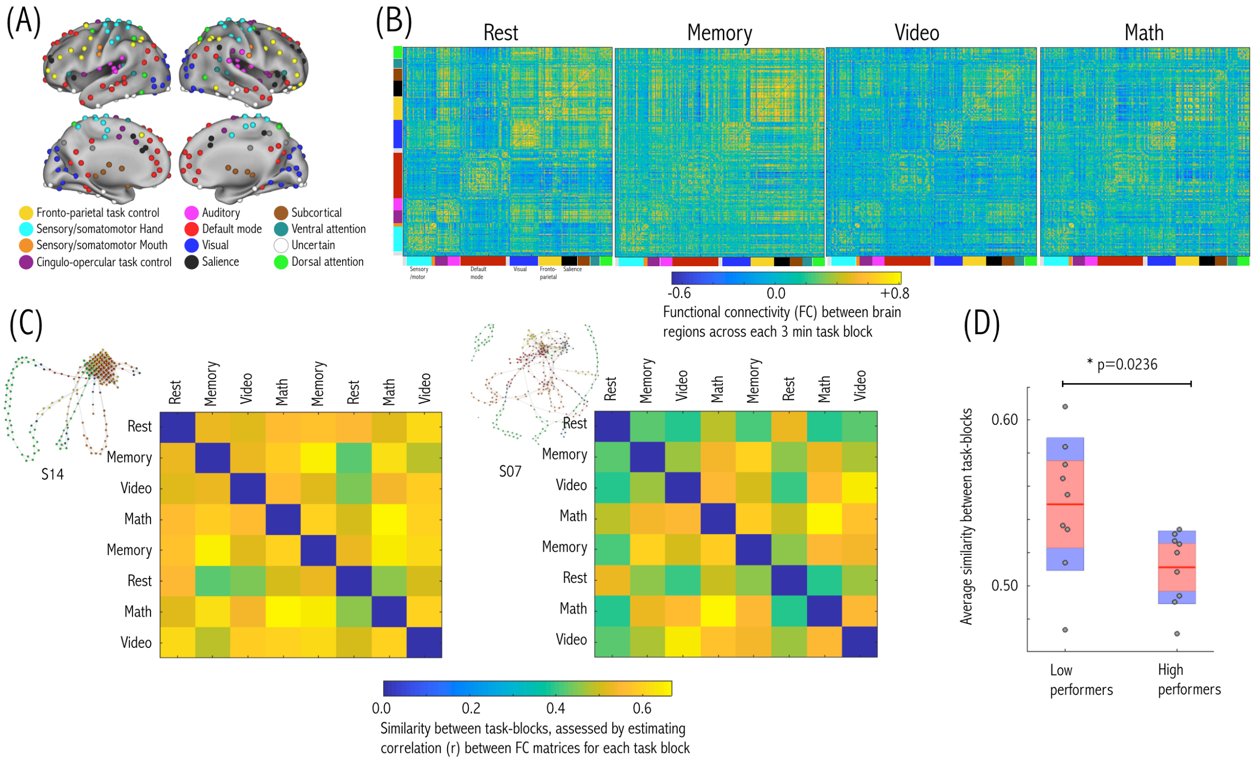

Supplementary Figure 12: Validation of a TDA-derived prediction that participants with higher task-performance evoked task-specific brain configurations as compared to participants with lower task-performance. Using the overall task performance, a median split was used to divide the participants into two groups (low- (n=9) and high-performers (n=9) with a median split at 86.9% accuracy). An independent and well-established brain parcellation scheme[2] was used to parcellate the brain into 264 brain regions (as shown in A). (B) Shows the whole-brain functional connectivity (FC) matrices for each task block for a representative participant (S01). (C) Shows similarity between FC matrices derived from different tasks for two representative participants (S14 – from the low-performance group and S07 – from the high-performance group). Please note that that the obvious value (=1) of the diagonal elements was zeroed-out. As descriptively evident, FC matrices from S14 were more similar between tasks as compared to FC matrices from S07. (D) Shows quantitative group-level comparison – average similarity between FC matrices for each participant was estimated and a two-sample t-test was run to compare the low-performance group from the high-performance group. A significant t-statistic ($t(16) = 2.50$, $p=0.0236$) was observed, such that participants in the low-performance group (compared to high-performance group) had higher average similarity between FC matrices derived from different task blocks.

Supplementary Table 1: Results from the weighted GLM analysis to find the neurophysiological basis of coreness in the shape graphs. Using a GLM analysis, weighted time frames were entered for each task separately (i.e., four explanatory variables). Two contrasts were run to examine brain regions that show positive as well as negative association with the coreness scores. The cluster-corrected ($Z>2.3$ and FWER $p<0.05$) group-level results are presented below (size of cluster, p-value and z-value, and location of the peak activation in each cluster).

		Cluster Index	Voxels	P	Z Value	Z-MAX X (mm)	Z-MAX Y (mm)	Z-MAX Z (mm)
Working Memory	*Positively associated with Coreness scores (CS)*	7	835	5.96E-08	4.03	48	24	33
		6	528	1.63E-05	3.71	-33	18	-3
		5	511	2.26E-05	4.19	45	-54	33
		4	427	0.000119	4.05	-6	3	51
		3	382	0.000304	3.95	30	18	0
		2	361	0.000476	4.48	-42	21	33
		1	331	0.000918	3.86	-33	-57	33
	Negatively associated with Coreness scores (CS)	4	5116	1.02E-28	4.32	33	-33	-36
		3	337	0.000804	3.42	-24	-54	69
		2	264	0.0043	3.79	6	51	-21
		1	178	0.0379	3.33	-6	63	21
Math	*Positively associated with Coreness scores (CS)*	2	351	0.000666	3.33	42	-54	33
		1	206	0.0196	3.57	9	12	48
	Negatively associated with Coreness scores (CS)	4	539	1.56E-05	3.36	6	-81	-30
		3	462	6.81E-05	3.16	-6	-42	-12
		2	192	0.0281	3.38	3	-12	45
		1	189	0.0304	3.56	63	-6	0
Video	*Positively associated with Coreness scores (CS)*	4	538	7.93E-06	3.85	48	-69	-27
		3	348	0.000431	3.79	-42	-66	-12
		2	175	0.0331	3.09	51	21	30
		1	167	0.0415	3.26	36	54	18
	Negatively associated with Coreness scores (CS)	1	211	0.0124	3.27	-12	-69	3

Supplementary References:

1. Woolrich, M. W., Behrens, T. E. J., Beckmann, C. F. & Smith, S. M. Mixture models with adaptive spatial regularization for segmentation with an application to FMRI data. *IEEE Trans Med Imaging* **24,** 1–11 (2005).
2. Power, J. D. *et al.* Functional network organization of the human brain. *Neuron* **72,** 665–678 (2011).
