# Supplementary material for: Towards a new approach to reveal dynamical organization of the brain using topological data analysis
Source: Nat Commun. 2018 Apr 11;9:1399. doi: 10.1038/s41467-018-03664-4 (PMC5895632; doi:10.1038/s41467-018-03664-4)
Supplement: Supplementary file 3 — Description of Additional Supplementary Files(PDF 4 kb) [file 41467_2018_3664_MOESM3_ESM.pdf]

Description of Additional Supplementary Files

File Name: Supplementary Movie 1
Description: A web-based interactive tool was developed to interact with the shape graphs. The tool is developed to interact with the shape graph while displaying the associated spatiotemporal information. The tool provides the spatial profile generated from the mixture modeling at each time frame (or TR). It also displays spatial correlation with known large-scale brain networks in real time. Additionally, the color and pie charts for each node can be changed in real time to display different pieces of information (e.g., hit/miss trials etc.). Lastly, the tool allows for generating temporal movies to review how spatial topographies change in time as the participant transitions from one task to another.
